# Percutaneous coronary intervention in insulin‐treated diabetic patients: A meta‐analysis

**DOI:** 10.1111/anec.12953

**Published:** 2022-04-25

**Authors:** Ying Ge, Daikun He, Yiru Shao, Lina Wang, Wei Yan

**Affiliations:** ^1^ 593949 Department of General Practice Jinshan Hospital of Fudan University Shanghai China; ^2^ 593949 Center of Emergency & Intensive Care Unit Jinshan Hospital of Fudan University Shanghai China

**Keywords:** diabetes mellitus, insulin, meta‐analysis, percutaneous coronary intervention, systematic review

## Abstract

**Background:**

This meta‐analysis of randomized controlled trials (RCTs) compared long‐term adverse clinical outcomes of percutaneous coronary intervention (PCI) in insulin‐treated diabetes mellitus (ITDM) and non‐ITDM patients.

**Methods:**

This is a meta‐analysis study. The PubMed and Embase databases were searched for articles on long‐term adverse clinical outcomes of PCI in ITDM and non‐ITDM patients. The risk ratios (RR) and 95% confidence intervals (CI) were calculated.

**Results:**

A total of 11 related RCTs involving 8853 DM patients were included. Compared with non‐ITDM patients, ITDM patients had significantly higher all‐cause mortality (ACM) (RR = 1.52, 95% CI: 1.25–1.85, *p*_heterogeneity_ = .689, *I* ^2^ = 0%), major adverse cardiac and cerebrovascular events (MACCE) (RR = 1.35, 95% CI: 1.18–1.55, *p*_heterogeneity_ = .57, *I* ^2^ = 0%), myocardial infarction (MI) (RR = 1.41, 95% CI: 1.16–1.72, *p*_heterogeneity_ = .962, *I* ^2^ = 0%), and stent thrombosis (ST) (RR = 1.75, 95% CI: 1.23–2.48, *p*_heterogeneity_ = .159, *I* ^2^ = 32.4%). No significant difference was found in the target lesion revascularization (TLR) and target vessel revascularization (TVR) between the ITDM and non‐ITDM groups.

**Conclusions:**

The results showed that ITDM patients had significantly higher ACM, MACCE, MI, and ST, compared with non‐ITDM patients.

## 1 METHODS

Diabetes mellitus (DM) has become one of the most important public health concerns of this century. Currently, over 366 million people worldwide have DM and this number is likely to double by 2030 (Guariguata et al., 2011; Shaw et al., 2010). Low‐ and middle‐income countries share the biggest burden of this disease, which accounts for about 80% of all cases (Zhang et al., 2010). Asia has the world's most rapid rise of T2DM population, with China and India as the two epicenters (Zheng et al., 2018). According to the national survey report in 2007, the prevalence of DM in China is 9.7%, with an estimated 92.4 million adults suffering from DM (Yang et al., 2010). In 2013, 11.6% of Chinese over the age of 18 years (about 1.14 million) reported having DM (Xu et al., 2013).

DM patients with coronary artery disease have an increased risk of cardiovascular events, and its prevalence is increasing (Ingelfinger & Jarcho, 2017). Insulin‐treated diabetes mellitus (ITDM) is known to be associated with a particularly high risk of cardiovascular events. These patients usually start using insulin in the later stages of DM, resulting in frequent associated comorbidities (Hamaty, 2011). ITDM patients have an increased all‐cause and cardiovascular mortality one year after percutaneous coronary intervention (PCI) and are at greater risk of adverse consequences (Noman et al., 2017; Q. Wang et al., 2018). However, the adverse clinical outcomes after PCI in ITDM and non‐ITDM patients remain unclear. So far, no meta‐analysis based on RCTs has been constructed. Therefore, we conducted this meta‐analysis to compare long‐term adverse clinical outcomes after PCI in a larger sample size of ITDM and non‐ITDM patients.

The present meta‐analysis was conducted according to the Preferred Reporting Items for Systematic Reviews and Meta‐analysis guidelines (Moher et al., 2009). Institutional Review Board approval was not required because this article is a meta‐analysis. The data come from published articles and do not require ethics approval.

### 1.1 Literature search

The Pubmed and Embase databases were systematically searched for articles reporting on adverse clinical outcomes in DM patients after PCI. The retrieval period was from the establishment of the database to December 2021. We used Mesh terms with the following search strategies: (“diabetes” OR “diabetes mellitus”) AND (“percutaneous coronary intervention” OR “PCI”) AND “insulin.” Language was restricted to English. We also searched references of the included articles to identify additional studies. Two investigators independently reviewed the citations.

### 1.2 Selection criteria

The inclusion criteria were as follows: (1) RCTs involving only DM patients after PCI; (2) studies having at least two groups, one group receiving treatment with insulin and another group receiving treatment without insulin; (3) studies providing long‐term (≥12 months) adverse outcomes; and (4) studies providing risk ratios (RRs) with 95% confidence intervals (CIs) or the ability to calculate these statistics from the data provided. Abstracts, case reports, conference presentations, editorials, and reviews were excluded.

### 1.3 Data extraction and quality assessment

Two investigators independently performed data extraction, and disagreements were settled by a third investigator. The inclusion and exclusion criteria were strictly followed in the process of literature screening. The following data were collected: first author's name, year of publication, country, gender, mean age, the number of patients with hypertension, the number of patients with dyslipidemia, sample size, and follow‐up time. The Cochrane Collaboration's risk‐of‐bias tool was used to evaluate the quality of randomized controlled trials (RCTs) for bias risk assessment (Higgins et al., 2011).

### 1.4 Statistical analysis

Stata ve.14.0^®^ licensed for StataCorp, College Station, TX, USA, was used for statistical analysis to quantitatively assess the mortality rate and complication rate of DM patients after PCI. The heterogeneity between the studies was analyzed by the chi‐squared test and was quantitatively determined by *I* ^2^. *I* ^2^ > 50% as evidence of heterogeneity. If the heterogeneity was significant, the random‐effects model was adopted. Otherwise, a fixed‐effects model was adopted. By individually removing each study for sensitivity analysis, the relative impact of each study on the comprehensive assessment was evaluated. Publication bias was assessed by visual inspection of the Begg's funnel plot symmetry and evaluation of the Egger's test (*p* < .05).

### 1.5 Patient and public involvement

Not applicable.

## 2 RESULTS

### 2.1 Study selection

**FIGURE 1:**
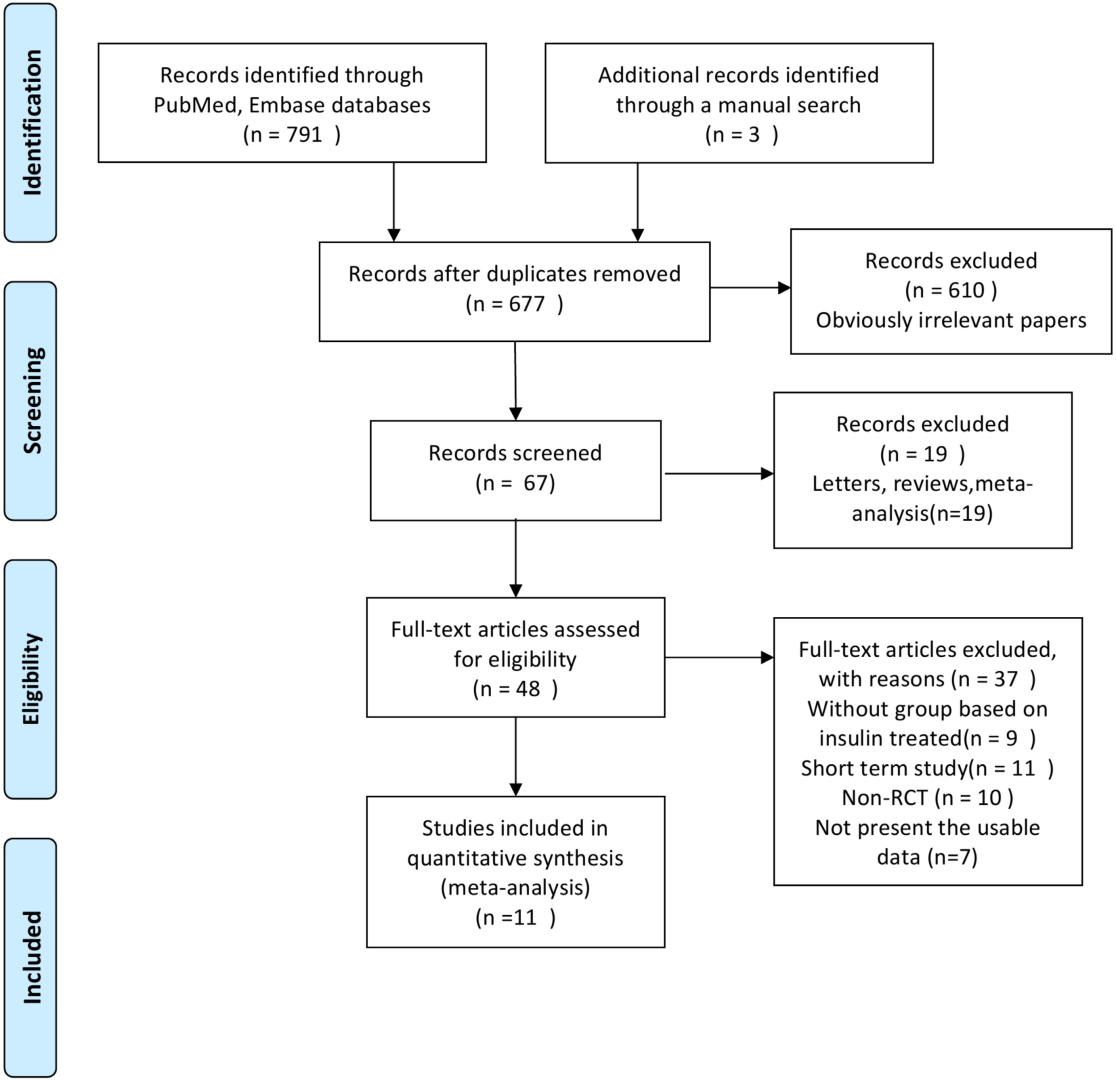
Flow chart of identification of studies

The database searches returned 677 records, of which 610 were excluded as irrelevant based on the title and abstract. The remaining studies were systematically evaluated, and full texts of 48 studies were retrieved. Thirty‐seven studies were excluded because of no insulin‐treated group (*n* = 9), short term study (*n* = 11), non‐RCTs (*n* = 10), and no relevant data (*n* = 7). Finally, 11 RCTs (Bangalore et al., 2016; Banning et al., 2010; Dangas et al., 2014; Hermiller et al., 2005; Kalkman et al., 2017; Kappetein et al., 2013; Kereiakes et al., 2010; Kirtane et al., 2008, 2009; Stone et al., 2011; Witzenbichler et al., 2011) were included in this analysis. Figure 1 shows the detailed search process.

### 2.2 Study characteristics

**FIGURE 2:**
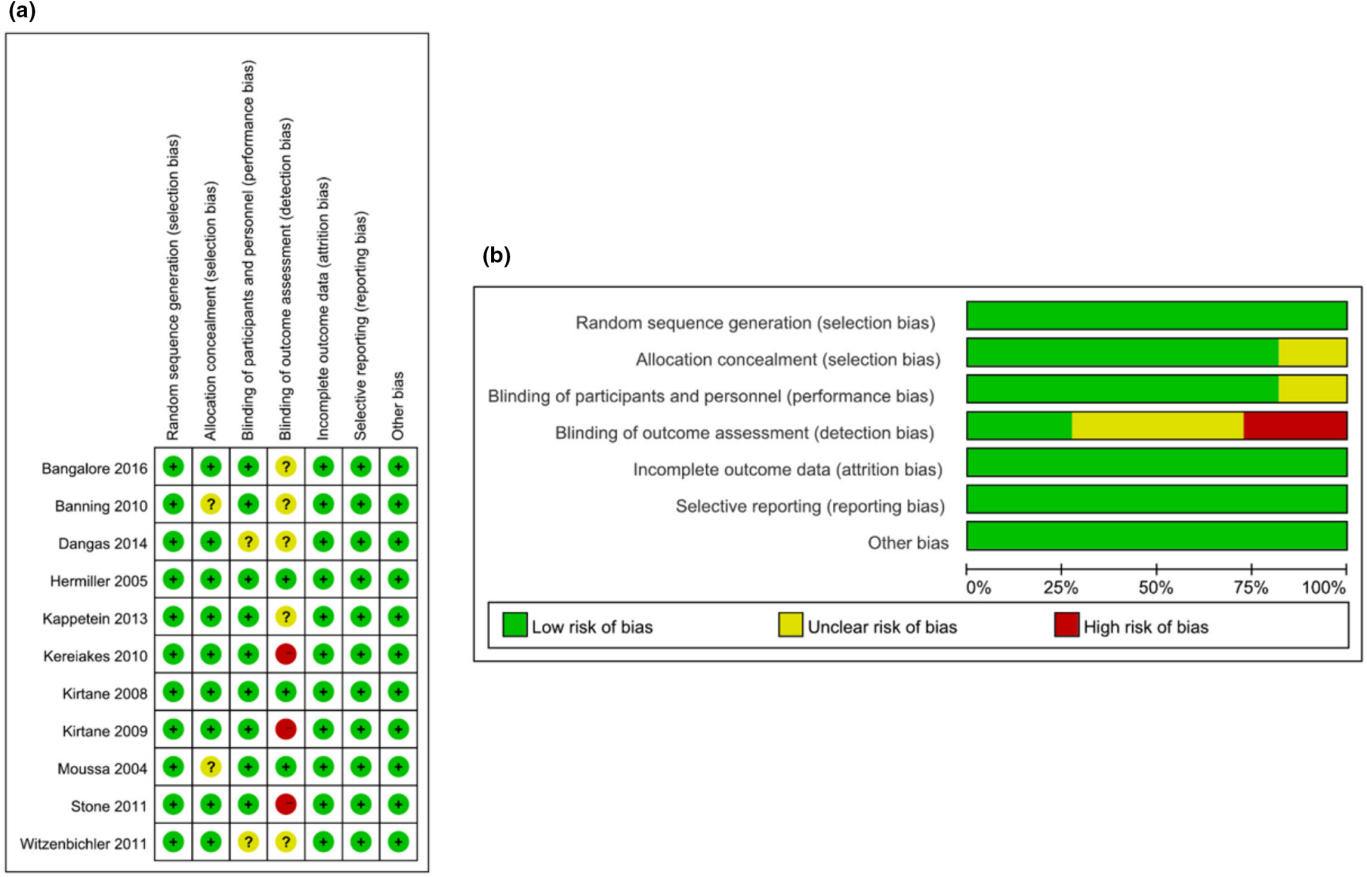
Risk‐of‐bias assessments for the randomized controlled trials included in the meta‐analysis. (a) Risk‐of‐bias summary; (b) Risk‐of‐bias graph. (+): Low risk of bias; (?): unclear risk of bias; (–): high risk of bias

Eleven eligible RCTs were selected, including 2881 ITDM and 5972 non‐ITDM patients. Among these studies, four were conducted in Europe, and 7 in North America. The follow‐up interval of these studies ranged from 12 to 60 months. The main features of the qualified studies are shown in Table 1. A summary of biases determined in each RCT is shown in Figure 2. All the included studies were of medium or high quality.

**TABLE 1:** Characteristics of the studies included in the meta‐analysis

Authors/year of publication	Country	Mean age (Y) IT/NIT	Male (%) IT/NIT	Hypertension (%) IT/NIT	Dyslipidemia (%) IT/NIT	Intervention		Follow‐up
						IT	NIT	
Hermiller/2005 (Hermiller et al., 2005)	USA	62.2/62.2	63.5/63.5	81.1/81.1	71.4/71.4	105	213	12 M
Kirtane/2008 (Kirtane et al., 2008)	USA	63/63	64.7/64.7	82.1/82.1	74/74	265	562	48 M
Kirtane/2009 (Kirtane et al., 2009)	USA	64/64	60.4/60.4	90.6/90.6	87.1/87.1	137	319	12 M
Banning/2010 (Banning et al., 2010)	UK	65.4/65.4	71/71	69.9/69.9	81.5/81.5	182	270	12 M
Kereiakes/2010 (Kereiakes et al., 2010)	USA	63.3/63.3	63.3/63.3	87/87	82.5/82.5	314	826	12 M
Stone/2011 (Stone et al., 2011)	USA	63.8/63.8	63.2/63.2	83.1/83.1	79.4/79.4	494	1375	24 M
Witzenbichler/2011 (Witzenbichler et al., 2011)	Italy	64.5/64.5	73.4/73.4	72.3/72.3	60.3/60.3	159	434	12 M
Kappetein/2013 (Kappetein et al., 2013)	Netherlands	65.4/65.4	71/71	70/70	82/82	89	142	60 M
Dangas/2014 (Dangas et al., 2014)	USA	62.6/63.2	61.3/76.5	87.5/83.2	NA	325	631	60 M
Bangalore/2016 (Kalkman et al., 2017)	USA	58.52/58.27	71/78.2	65.6/67.1	NA	747	1083	12 M
Kalkman/2017 (Bangalore et al., 2016)	Netherlands	64.8/68.6	67.2/72.9	79.7/79.7	NA	64	117	12 M

Abbreviations: IT, insulin‐treated; M, months; NA, not available; NIT, Non‐insulin‐treated; Y, years.

### 2.3 Long‐term adverse clinical outcomes

**FIGURE 3:**
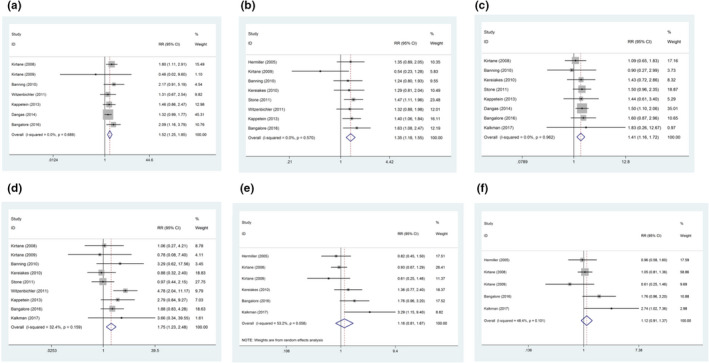
Forest plots showing adverse clinical outcomes in ITDM patients and non‐ITDM patients after PCI during the long‐term follow‐up. (a) ACM; (b) MACCE; (c) MI; (d) ST; (e) TLR; (f) TVR

*All*‐*cause mortality (ACM)*: ACM was reported in 7 studies. Compared with non‐ITDM patients, ITDM patients had significantly higher ACM (RR = 1.52, 95% CI: 1.25–1.85, *p*_heterogeneity_ = .689, *I* ^2^ = 0%), which is shown in Figure 3a.

*Major adverse cardiac and cerebrovascular events (MACCE)*: MACCE was reported in 8 studies. Compared with non‐ITDM patients, ITDM patients had significantly higher MACCE (RR = 1.35, 95% CI: 1.18–1.55, *p*_heterogeneity_ = .570, *I* ^2^ = 0%), which is shown in Figure 3b.

*Myocardial infarction (MI)*: MI was reported in 8 studies. Compared with non‐ITDM patients, ITDM patients had significantly higher MI (RR = 1.41, 95% CI: 1.16–1.72, *p*_heterogeneity_ = .962, *I* ^2^ = 0%), which is shown in Figure 3c.

*Stent thrombosis (ST)*: ST was reported in 9 studies. Compared with non‐ITDM patients, ITDM patients had significantly higher ST (RR = 1.75, 95% CI: 1.23–2.48, *p*_heterogeneity_ = .159, *I* ^2^ = 32.4%), which is shown in Figure 3d.

*Target lesion revascularization (TLR)*: TLR was reported in 6 studies. TLR showed no significant difference (RR = 1.16, 95% CI: 0.81–1.67, *p*_heterogeneity_ = .058, *I* ^2^ = 53.2%) between the ITDM and non‐ITDM patients, which is shown in Figure 3e.

*Target vessel revascularization (TVR)*: TVR was reported in five studies. TVR showed no significant difference (RR = 1.12, 95% CI: 0.91–1.37, *p*_heterogeneity_ = .101, *I* ^2^ = 48.4%) between the ITDM and non‐ITDM patients, which is shown in Figure 3f.

### 2.4 Sensitivity analysis

**FIGURE 4:**
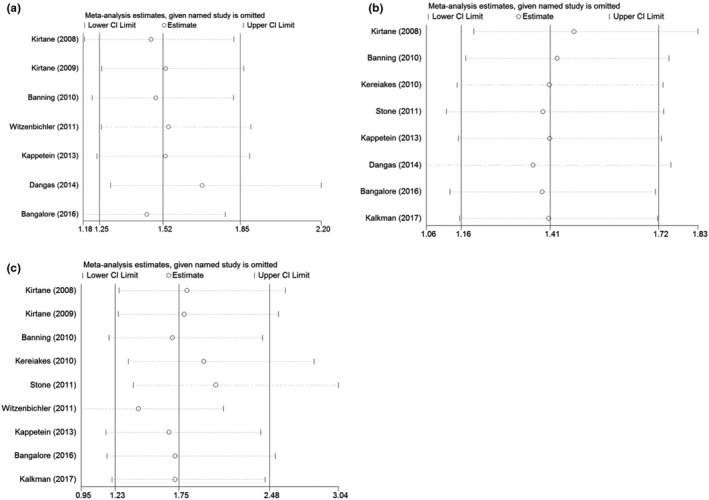
Sensitivity analysis of the influence of individual studies on pooled results. (a) ACM; (b) MI; and (c) ST

The sensitivity analysis was used to evaluate the impact of a single dataset on the summary results by sequentially deleting each eligible study. As shown in Figure 4a–c, the overall statistical significance remained the same after sequentially deleting each study, indicating that the results were statistically robust.

### 2.5 Publication bias

**FIGURE 5:**
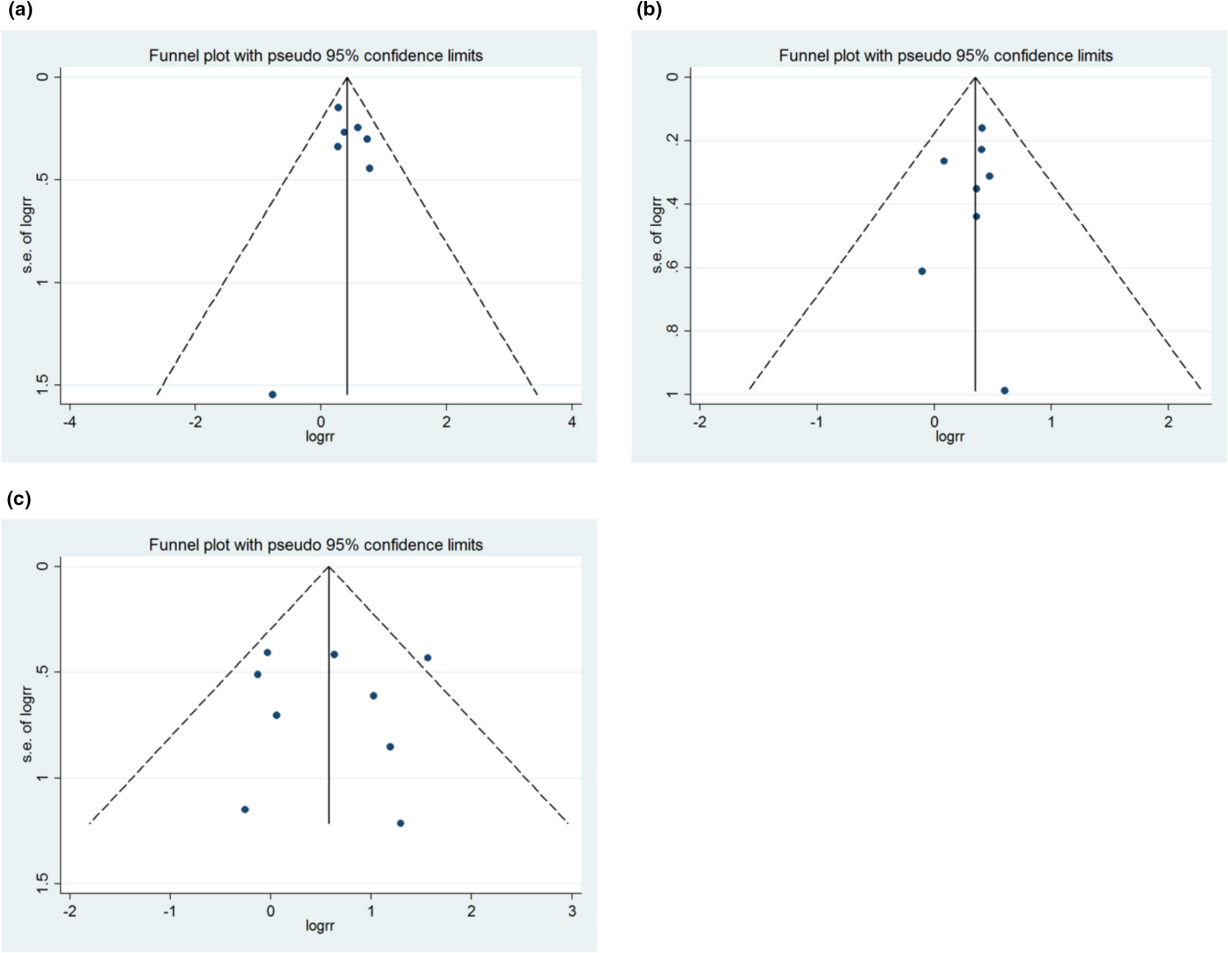
Funnel plot for publication bias test. (a) ACM; (b) MI; and (c) ST. Each point represents a separate study for the indicated association

The Egger's regression test showed no signs of asymmetric distribution in all‐cause mortality (Begg's test *p* = 1; Egger's test *p* = .739) (Figure 5a), myocardial infarction (Begg's test *p* = .266; Egger's test *p* = .564) (Figure 5b), and stent thrombosis (Begg's test *p* = .602; Egger's test *p* = .947) (Figure 5c).

## 3 DISCUSSION

DM is the third most common comorbidity among patients with cardiovascular disease, and 20–30% of patients with ischemic heart disease receive PCI treatment (Ritsinger et al., 2015). ITDM accounts for one‐fourth of all DM patients, usually with a longer course of disease, a high incidence of comorbidities, and poor blood sugar control (Bangalore et al., 2016). Previous studies have shown that ITDM patients have significantly worse clinical outcomes after PCI than non‐ITDM patients; however, the results were inconsistent (Bundhun et al., 2016; Dangas et al., 2014; Kappetein et al., 2013). This meta‐analysis compared long‐term adverse clinical outcomes of PCI in ITDM and non‐ITDM patients. A total of 11 related RCTs comprising 8853 patients were included. Compared with non‐ITDM patients, ITDM patients had significantly higher ACM, MACCE, MI, and ST. No significant difference in the TLR and TVR was found between the ITDM and non‐ITDM patients.

The adverse clinical outcomes of PCI in ITDM and non‐ITDM patients have been investigated in previous meta‐analysis. However, no meta‐analysis was conducted only based on RCTs. The current meta‐analysis included the ITDM and non‐ITDM patients, which involved a total of 8853 DM patients (ITDM: 2881; non‐ITDM: 5972) from 11 RCTs. Recently, Hassan et al. (2021) conducted a comprehensive meta‐analysis of the adverse clinical outcomes of PCI in ITDM and non‐ITDM patients. Compared with Bundhun's study, we only focused on RCTs and long‐term studies. Furthermore, we removed a retrospective cohort study (Beneduce et al., 2020) that was used as an RCT study by Hassen et al. During the two‐year follow‐up, Jiang et al. (2017) found that patients with ITDM were more susceptible to stent thrombosis, TVR, and MACCE. Jain et al. (2010) showed that insulin therapy was not statistically correlated with the increased tendency of stent thrombosis, although ITDM is a high‐risk factor for other cardiovascular adverse events. In DM patients, insulin resistance is associated with harmful biological processes, such as impaired angiogenesis of nitric oxide, and elevated levels of endothelin‐I and angiotensin‐II (Seabra‐Gomes, 2006). Insulin has both pro‐atherogenic and anti‐atherogenic properties, which alter the risk of cardiovascular events depending on the presence of insulin resistance and hyperinsulinemia (Wang et al., 2004). In terms of cumulative risk of adverse events, ACM, MACCE, MI, and ST were significantly increased in DM patients treated with insulin, when compared with non‐ITDM patients. The observation that ITDM patients had significantly higher serious cardiovascular events might have clinical implications in selecting the coronary revascularization strategy for these patients.

Meanwhile, some limitations in this meta‐analysis should be noted. First, there was wide variability in baseline characteristics of the included studies. Second, we searched the databases for articles only written in English, which may have excluded articles written in other languages. Third, we only included RCTs and removed observational studies. Observational studies have a high risk of selection bias and confounding by indication. Fourth, several studies of small sample sizes may reduce the statistical power. Fifth, acute coronary syndrome (ACS) includes acute ST‐segment elevation myocardial infarction (STEMI), acute non ST‐segment elevation myocardial infarction (NSTEMI), and unstable angina pectoris (UA). There are some differences in the treatment strategy and prognosis of different types of ACS. Unfortunately, we cannot find available information from the included literatures for subgroup analysis. Finally, the results were based on unadjusted assessment of RRs, which might influence the results.

According to this study, ITDM patients had significantly higher ACM, MACCE, MI, and ST, compared with non‐ITDM patients.

## CONFLICT OF INTEREST

The authors have stated explicitly that there are no conflicts of interest in connection with this article.

## AUTHOR CONTRIBUTIONS

Dai‐KunHe conceived and coordinated the study, designed, performed, and analyzed the experiments, and wrote the article. Yi‐Ru Shao, Ying Ge, Lina Wang, and Wei Yan carried out the data collection, data analysis, and revised the article. All the authors reviewed the results and approved the final version of the manuscript.

## ETHICS APPROVAL

Not applicable.

## Data Availability

The data set supporting the results of this article is included within the article.
